# Disruption of brain regional homogeneity and functional connectivity in male NAFLD: evidence from a pilot resting-state fMRI study

**DOI:** 10.1186/s12888-023-05071-6

**Published:** 2023-08-29

**Authors:** Kun Shu, Xinjian Ye, Jiawen Song, Xiaoyan Huang, Shihan Cui, Yongjin Zhou, Xiaozheng Liu, Lu Han, Zhihan Yan, Kun Liu

**Affiliations:** 1 Department of Radiology, The Second Affiliated Hospital and Yuying Children’s Hospital of Wenzhou Medical University, Wenzhou, 325027 China; 2 Philips Healthcare, Shanghai, China

**Keywords:** Non-alcoholic fatty liver disease, Cognition, rs-fMRI, ReHo, Functional connectivity

## Abstract

**Background:**

The neurophysiological mechanisms underlying cognitive deficits in non-alcoholic fatty liver disease (NAFLD) remain unknown. Cognitive changes may be caused by brain alterations in neural activity and functional connectivity (FC).

**Aim:**

This study aims to investigate the alterations between spontaneous brain neural activity and FC in male NAFLD patients and the relationship of neural activity with cognitive performance.

**Methods:**

In this prospective study, 33 male pre-cirrhosis NAFLD subjects and 20 male controls matched for age, education level, and body mass index. All participants underwent resting-state functional magnetic resonance imaging scans and neuropsychological examinations. Regional homogeneity (ReHo) analysis was used to investigate the brain function in NAFLD, and regions with significantly altered ReHo were selected as seeds for subsequent FC analysis. Partial correlation analysis was used to assess the relationships between altered ReHo measures and cognitive performance indicators.

**Results:**

Compared with the controls, the NAFLD patients showed increased ReHo in the opercular part of the right inferior frontal gyrus (IFGoperc) and decreased ReHo in the right middle frontal gyrus (MFG) and left superior parietal gyrus (SPG). The subsequent FC analysis showed increased FC between these regions (right IFGoperc, right MFG, and left SPG) and nodes of the default mode network (DMN) (such as left supraMarginal, left median cingulate and paracingulate gyri, left precuneus, orbital part of left medial frontal gyrus, and bilateral posterior cingulate gyrus). In addition, significant positive correlations were observed between NAFLD patients’ clock drawing test scores and altered ReHo in prefrontal cortices (right IFGoperc and right MFG).

**Conclusion:**

Before developing cirrhosis, NAFLD patients showed altered neural activity in several brain regions and altered FC between the salience network and DMN. These alterations could potentially be a compensatory mechanism to maintain cognitive function in pre-cirrhosis NAFLD patients.

**Supplementary Information:**

The online version contains supplementary material available at 10.1186/s12888-023-05071-6.

## Introduction

Non-alcoholic fatty liver disease (NAFLD), ranging from simple hepatic steatosis to cirrhosis, is the leading cause of chronic liver disease and affects more than a quarter of the global population [1, 2]. Recent studies have shown that NAFLD is accompanied by cognitive impairments, including deficits in memory, execution, and visuospatial abilities [3, 4]. In the advanced stage of NAFLD, hepatic encephalopathy results from liver cirrhosis, causing a decline in brain function and cognition [5]. In fact, patients with NAFLD have brain damage before the cirrhosis stage [5]. Previous studies have reported several possible pathophysiological mechanisms of cognitive dysfunction caused by NAFLD, including neuroinflammation, accumulation of ammonia, and endothelial dysfunction [3]. However, the mechanisms underlying NAFLD-related cognitive impairment remain unclear and require further investigation.

Brain structural and functional changes may contribute to understanding the mechanism of cognitive impairment in NAFLD. Structural magnetic resonance imaging (MRI) studies demonstrate that NAFLD patients with lower MoCA scores have significantly reduced cerebral grey and white matter volumes in the brain and that MoCA scores correlate with gray and white matter volumes [6]. A Framingham study had similar findings [7]. These findings indicate that changes in cerebral structure contribute to the development of cognitive impairments in NAFLD. Using functional MRI, the CARDIA study found that NAFLD patients have lower gray matter cerebral blood flow than the controls and that NAFLD is negatively associated with brain health [8]. It suggests that altered cerebral perfusion may be another mechanism underlying cognitive dysfunction in NAFLD. However, these studies contain numerous uncontrolled confounding variables, including obesity and diabetes. Additionally, the absence of pathological diagnosis complicates the interpretation of the results. Resting-state functional MRI (rs-fMRI) is a reliable measure of blood oxygen concentration and has been extensively used to explore the mechanisms of cognitive impairment associated with hepatic cirrhosis [9–12]. Previous rs-fMRI studies have shown changes in neural activity in patients with cirrhosis, involving brain regions including the thalamus, frontal, temporal, and occipital lobes [13].[12]. These studies focused on patients with cirrhosis whose etiology was not limited to non-alcoholic cirrhosis, which may contribute to the inconsistency of their results. To date, there is relatively limited studies in rs-fMRI for pre-cirrhotic NAFLD patients.

Regional homogeneity (ReHo) is a robust rs-fMRI method for investigating the similarity or coherence of intraregional low-frequency (< 0.08 Hz) spontaneous blood oxygen level-dependent (BOLD) signal fluctuations in the voxel-wise analysis of the whole brain [15]. Functional connectivity (FC) is an rs-fMRI indicator for the temporal correlation of spontaneous neural activity between brain regions and is used to investigate brain network mechanisms [16]. Both methods are widely used in liver disease and other fields [12, 17–19]. ReHo and FC indicators significantly correlate with cognitive assessments [12, 18–20], suggesting an underlying neurophysiological mechanism of cognitive impairment.

In the present proof-of-concept pilot study, we investigated the consistency of intraregional spontaneous brain neural activity and FC in pre-cirrhosis male NAFLD patients by using ReHo and seed-based FC rs-fMRI techniques. Including only male participants was intended to eliminate hormonal effects on brain function [21]. We hypothesized that pre-cirrhosis male NAFLD patients have altered spontaneous neural activity and that these alterations are associated with neurocognitive performance. Additionally, the relationship between altered neural activity and pathological features of NAFLD was investigated.

## Materials and methods

### Subjects

In this prospective study, we enrolled 33 male subjects (mean age 41.7 years) with biopsy-proven NAFLD and 20 male controls (mean age 44.0 years) with matched age, education level, and BMI. Participants were collected between July 2017 and May 2019 from the First Affiliated Hospital and the Second Affiliated Hospital of Wenzhou Medical University. The enrollment criteria for the NAFLD group were: 1): right-handed Chinese men; 2) diagnosed with NAFLD by biopsy. The controls were defined as hepatic MRI-derived proton density fat fraction (MRI-PDFF) < 6.4% (fatty liver was diagnosed while MRI-PDFF ≥ 6.4%) [22]. In both NAFLD and control group, subjects were excluded for any of the following reasons: hepatitis B or hepatitis C virus infection; type II diabetes mellitus (T2DM) (fasting glucose level ≥ 7.0 mmol/L, a previous diagnosis of diabetes or treatment with any anti-hyperglycemic drugs) [23]; excessive alcohol consumption (consuming more than 14 alcoholic drinks per week); a history of psychiatric disorders or anti-psychotic treatments; other causes of chronic liver disease; and MRI contraindication. Fasting venous blood was collected on the same day as the liver biopsy. From blood samples, levels of fasting glucose, total bilirubin, alanine aminotransferase (ALT), aspartate aminotransferase (AST), alkaline phosphatase, gamma-glutamyltransferase (GGT), total cholesterol, triglycerides, high-density lipoprotein-cholesterol (HDL-cholesterol), and low-density lipoprotein-cholesterol (LDL-cholesterol) were measured. This study was approved by the ethics committees of the First Affiliated Hospital and the Second Affiliated Hospital of Wenzhou Medical University and all participants gave written informed consent.

### Pathological assessment of NAFLD

The pathology specimens for ultrasound-guide percutaneous liver biopsy were scored according to a widely approved histological scoring system by an experienced histopathologist blinded to the clinical and biochemical data of the patients [24, 25]. Liver biopsies assessment encompasses steatosis (grade 0 – 3), hepatocellular ballooning (grade 0 – 2), lobular inflammation (grades 0 – 3), and fibrosis (stages 0 – 4). Liver steatosis was defined as ≥ 5% of steatotic hepatocytes on the pathological specimen. The histologic NAFLD activity score (NAS) was calculated as the unweighted sum of hepatic steatosis, hepatocellular ballooning, and lobular inflammation scores [24]. According to the histological score of NAS, definite non-alcoholic steatohepatitis (NASH) was characterized by a NAS score ≥ 5. Conversely, scores of < 3 were ?not NASH?, and scores of 3 and 4 implied the possibility of NASH (indeterminate NASH) [24].

### Neurocognitive tests

Neurocognitive assessments were performed by an experienced neurologist who was blinded to the subjects’ information. The scales included the Mini-Mental State Examination (MMSE) for general mental status, the Rey-Osterreith Complex Figure Tests (CFT-Copy, CFT-Recall) for working memory and execution, the Digital Span Test (DST-Forward, DST-Backward) for attention, the Trial Making Test (TMT-A, TMT-B) for attention and execution, the Clock Drawing Test (CDT) for visuospatial function, and the Auditory Verbal Learning Test (AVLT-Immediate recall, AVLT-Delayed recall) for verbal memory [26, 27]. The neurocognitive assessments were completed within 24 h from the MRI scan. The detailed are shown in Supplementary Materials.

### MR image acquisition

MR images of all subjects were obtained on a 3.0 Tesla MR system (Discovery MR750, GE Healthcare) scanner equipped with an 8-channel phased array head coil. Rs-fMRI data were acquired using an echo-planner imaging sequence with the following parameters: repetition time (TR) = 2000 ms; echo time (TE) = 30 ms; flip angle (FA) = 90°; matrix size = 64 × 64; field of view (FOV) = 220 × 220 mm; slice thickness = 1.0 mm, 35 axial slices with 1 mm slice gap, acquiring 185 volumes. Three-dimensional T1-weighted structural images were acquired using a whole-brain sagittal spoiled gradient echo sequence with the following parameters: TR = 7.7 ms; TE = 3.4 ms; inversion time = 450 ms; FA = 12°; matrix size = 256 × 256; FOV = 256 × 256 mm, slice thickness = 1 mm and 188 sagittal slices. During the MRI scan, participants were instructed to lie still with closed eyes but not fall asleep.

### MRI-PDFF acquisition

A breath-hold (21 s) three-dimensional iterative decomposition of water and fat with echo asymmetry and least squares estimation quantification (IDEAL-IQ) sequence was performed to quantify hepatic fat fraction (MRI-PDFF) for all participants. The parameters were listed as follows: TR = 6.4 ms, TE = Min Full, FA = 3°, matrix size = 160 × 160; FOV = 35 × 24 cm; bandwidth = 111.11 kHz, 24 axial slices with 10 mm slice gap. MRI-PDFF measurement was completed by a senior abdominal radiologist blinded to the subjects’ information. The Image J software (U.S. National Institutes of Health) was used to process IDEAL-IQ images to automatically acquire hepatic PDFF images. Three regions of interest (ROI) with 150 mm^2^ in the area were placed at different locations in the right lobe of the liver, avoiding the adjacent structures and the main vessel, and the average of the three ROIs was considered as the result of MRI-PDFF.

### Rs-fMRI pre-processing

The rs-fMRI data were pre-processed using the DPABI toolbox v6.0 (www.rfmri.org). The first ten scan volumes were removed. The process was followed by section timing correction, realignment, and registration to individual structural T1 images. The gray matter, white matter, and cerebrospinal fluid were segmented. Then images were spatially normalized into the standard Montreal Neurological Institute (MNI) template with a resampling voxel size of 3 × 3 × 3 mm^3^. Band-pass filtering (0.01–0.08 Hz) was used to reduce the effect of high-frequency noise and low-frequency drift, and then linear trends were removed. White matter signal and cerebrospinal fluid signal were regressed out by Friston’s 24-parameter [28].

No participants were excluded from this study because head motion exceeded 3 mm translation or 3° rotation. There was no significant difference in the mean relative displacements of head motion between the two groups (t = -0.319, *p* = 0.751). In addition, volumes with mean framewise displacement (FD) > 0.2 mm, as well as two forward and one back of those volumes, were deleted to further reduce the influence of head movement on subsequent data analysis [29].

### ReHo analysis

ReHo analysis was performed using the DPARSF software. Individual ReHo map of each participant was obtained by calculating Kendall’s coefficient concordance (KCC) of the time series of a given voxel and its 26 neighbor voxels in a voxel-wise manner [15]. The ReHo value of a given voxel represented the regional temporal synchronization degree within a neighbor voxel cluster. Then the ReHo value was divided by the global mean KCC value in each subject to improve the ReHo value’s normality and reliability. Finally, the processed ReHo maps were smoothed with a Gaussian kernel of 6 × 6 × 6 mm.

### Seed-based functional connectivity analysis

Seed-based FC analysis was conducted based on the ReHo results. Regions showing significantly altered regional, temporal synchronization (ReHo value) between NAFLD and the control group were considered as ROI for subsequent FC analysis. For each subject, a reference time series was obtained by averaging the time series of voxels within each seed ROI. Pearson’s correlation coefficient was then calculated between the reference time series of each seed ROI and the time curse of all other brain voxels. Finally, Fisher’s z-transformation was applied to improve normality.

### Statistical analysis

Comparisons of demographic information (age, BMI, educational level), biochemical indicators, and neurocognitive assessments between NAFLD and control group were performed using SPSS27.0 software. Continuous variables were analyzed using the independent two-sample t-test or the Mann-Whitney U test, and *p* < 0.05 was considered as a significant difference. We compared the significance of the changes in ReHo and FC metrics between groups by using a voxel-based independent two-sample t-test with age, BMI, educational level, and mean FD as covariates. False discovery rate (FDR) correction was applied to multiple comparison corrections with a significance threshold of *p* < 0.05 and cluster size > 25.

To increase the ease of interpretation of the scores across cognitive tests with varying scales and score meanings, neurocognitive test scores were converted into standard z-scores (calculated based on the sample distribution). Additionally, to evaluate the intrinsic relationship between aberrant brain neural activity and neurocognitive performance in both NAFLD and control groups, ReHo values with significant differences between the two groups were extracted. In order to regress out the effect of age, BMI, and educational level, simple linear regression was performed to calculate the residuals for ReHo values and z-transformed cognitive scores. Pearson correlation analysis was used for these residuals. Spearman correlation analysis explored the relationship between altered neural activity and pathological features (liver MRI-PDFF, NAS, and NASH). Bonferroni correction was performed for multiple comparisons. *P* < 0.05 was considered a significant difference.

## Results

### Clinical and neurocognitive characteristics

The clinical characteristics of the two groups and the pathology of the liver in the NAFLD group are shown in Table 1. The two groups did not differ significantly in age (*p* = 0.12), BMI (*p* = 0.163), or education level (*p* = 0.072). However, the NAFLD patients had significantly higher AST (*p* = 0.012), GGT (*p* = 0.004), triglycerides (*p* = 0.023), and MRI-PDFF (*p* < 0.01) levels, as well as significantly lower HDL-cholesterol (*p* = 0.036), compared to the controls.

**Table 1:** Demographic, biochemical and pathological information with NAFLD compare to Controls

	NAFLD (n = 33)	Control (n = 20)	*p* value
**Demographics**			
Age, years	41.7 ± 4.9	44.0 ± 5.4	0.12
BMI, kg/m^2^	26.6 (24.7, 28.9)	25.8 (23.6, 27.4)	0.163
Education level, years	9 (6, 12)	9.0 (9.0, 15.8)	0.072
**Biochemical Measurements**			
Fast glucose, mmol/L	5.0 (4.7, 5.4)	5.1 (4.7, 5.5)	0.613
Total bilirubin, µmol/L	15.0 (11.5, 21.0)	15.6 (12.63, 20.0)	0.538
Alanine aminotransferase, U/L	47 (36, 84)	30.0 (21.2, 73.3)	0.071
Aspartate aminotransferase, U/L	32.0 (26.0, 47.5)	24.0 (20.3, 33.4)	0.012*
Alkaline phosphatase, U/L	81.5 ± 18.9	76.4 ± 22.5	0.382
Gamma-Glutamyltransferase, U/L	57.0 (36.5, 98.5)	35.0 (22.0, 49.5)	0.004*
Total cholesterol, mmol/L	4.94 ± 1.11	4.67 ± 0.73	0.339
Tryglicerides, mmol/L	2.15 ± 0.98	1.56 ± 0.72	0.023*
HDL-cholesterol, mmol/L	0.99 ± 0.14	1.15 ± 0.29	0.036*
LDL-cholesterol, mmol/L	3.10 (2.43, 3.62)	2.86 (2.24, 3.50)	0.48
**Liver Pathology**			
Hepatic PDFF, %	9.29 (5.66, 11.84)	3.83 (2.70, 4.70)	< 0.01*
Steatosis grade, n (%)			
S1	19 (57.6%)		
S2	6 (18.2%)		
S3	8 (24.2%)		
Inflammation grade, n (%)			
L0	1 (3.0%)		
L1	27 (81.8%)		
L2	5 (15.2%)		
Ballooning degeneration grade, n (%)			
B0	9 (27.3%)		
B1	18 (54.5%)		
B2	6 (18.2%)		
Fibrosis stage, n (%)			
F0	7 (21.2%)		
F1	25 (75.8%)		
F2	1 (3.0%)		
NAS			
NAS-2	6 (18.2%)		
NAS-3	11 (33.3%)		
NAS-4	7 (21.2%)		
NAS-5	5 (15.2%)		
NAS-6	4 (12.1%)		
NASH disease status			
NAFL	6 (18.2%)		
Borderline NASH	18 (54.5%)		
Define NASH	9 (27.3%)		

Notes: Values are mean (± standard deviation), median (interquartile range) or n (%)

Abbreviations: BMI, body mass index; HDL, high-density lipoprotein; LDL, low-density lipoprotein; PDFF, proton density fat fraction; NAFL: non-alcoholic fatty liver; NAS, NAFLD activity score. NASH, non-alcoholic steatohepatitis

Table 2 summarizes the z-transformed neurocognitive measurements of the two groups. The NAFLD group had significantly higher AVLT scores (AVLT-Immediate recall, *p* = 0.016; AVLT-Delayed recall, *p* = 0.018) than the control group. However, other neurocognitive data observed no significant differences between the two groups. Original neurocognitive measurements are summarized in Table S1.

**Table 2:** Neurocognitive measurements with NAFLD compare to the Controls

	NAFLD (n = 33)	Control (n = 20)	*p* value
MMSE	-1.15 ± 1.09	0.19 ± 0.82	0.616
AVLT-Immediate recall	0.22 ± 1.15	-0.36 ± 0.53	0.016*
AVLT-Delayed recall	0.25 ± 1.00	-0.41 ± 0.86	0.018*
CDT	0.01 ± 0.93	-0.02 ± 1.13	0.818
TMT-A	-0.15 ± 0.75	0.24 ± 1.30	0.233
TMT-B	-0.12 ± 0.84	0.21 ± 1.23	0.367
CFT-Copy	0.03 ± 0.87	-0.05 ± 1.20	0.946
CFT-Recall	0.19 ± 1.02	-0.32 ± 0.90	0.073
DST-Forward	0.19 ± 0.84	-0.31 ± 1.18	0.146
DST-Backward	-0.07 ± 1.04	0.11 ± 0.95	0.337

Notes: Z-transformed neurocognitive scores are expressed as mean ± standard deviation

Abbreviations: MMSE, Mini-Mental State Examination; AVLT, Auditory Verbal Learning Test; CDT, Clock Drawing Test; TMT, Trail Making Test; CFT, Rey-osterrieth Complex Figure Test; DST, Digital Span Test

### Differences in static ReHo

**Fig. 1:**
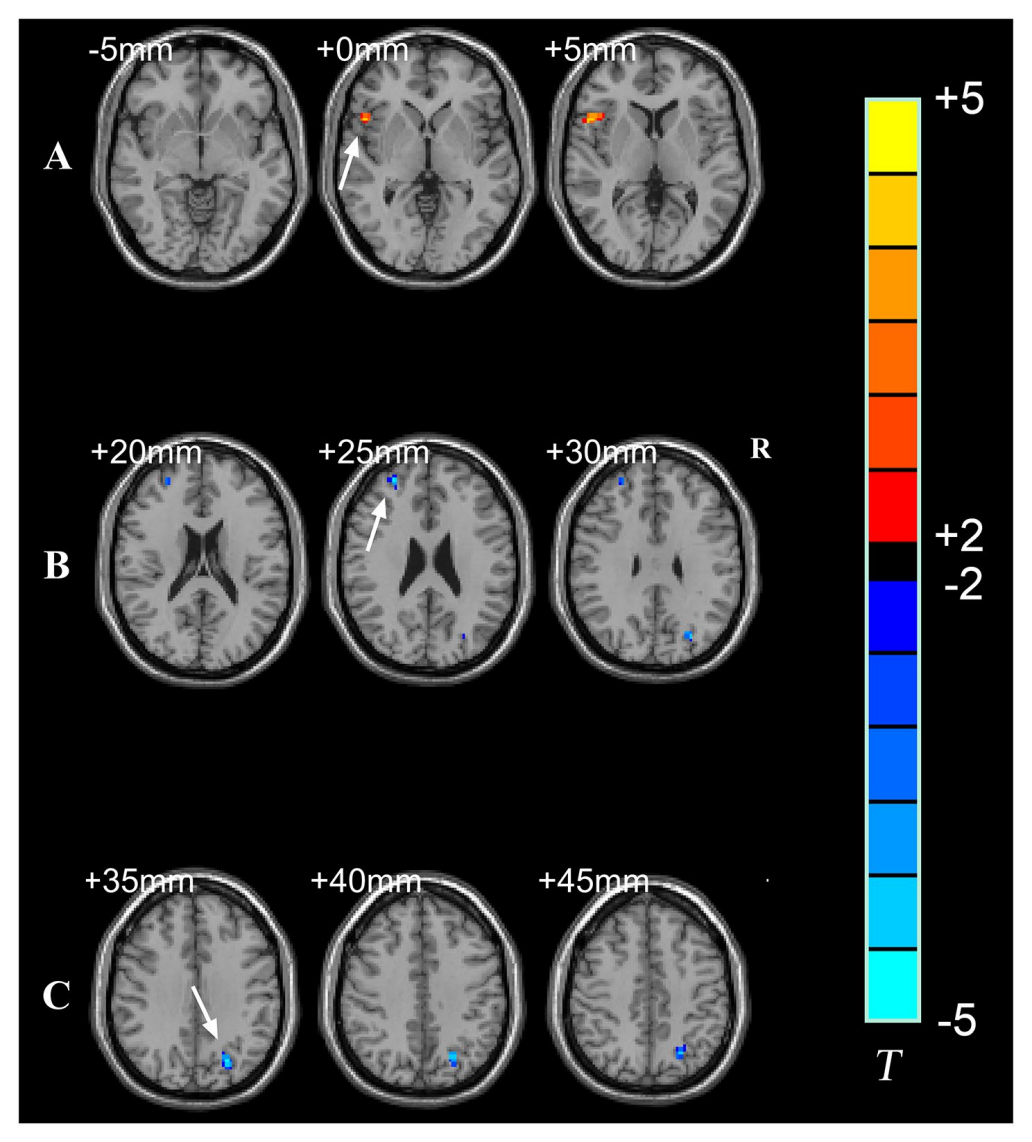
Brain regions with significantly altered regional homogeneity (ReHo) of **(A)** the opercular part of the right inferior frontal gyrus; **(B)** the right middle frontal gyrus; and **(C)** the left superior parietal gyrus in the NAFLD group compared with the control group. Voxel-based independent two-sample t-test analyses were performed with a significant threshold *p* < 0.05 (FDR correction) and cluster size > 25

The NAFLD group displayed significantly higher ReHo in the opercular part of the right inferior frontal gyrus (IFGoperc) and significantly lower ReHo in the right middle frontal gyrus (MFG) and left superior parietal gyrus (SPG) when compared with the control group (Fig. 1). The detailed results are summarized in Table 3.

**Table 3:** Regions with ReHo significant differences in NAFLD and controls

Brain regions	Cluster size (mm^3^)	Peak coordinate (MNI)			Peak *t* value
		X	Y	Z	
Right inferior frontal gyrus, opercular part (IFGoperc)	40	51	18	3	4.52
Right middle frontal gyrus (MFG)	25	30	51	24	-4.11
Left superior parietal gyrus (SPG)	56	-24	-66	42	-4.16

Notes: Peak coordinate represents the locations of peak in the Montreal Neurological Institute (MNI) space and the t value means the increase (*t* > 0) or decrease (*t* < 0) of regional homogeneity in NAFLD compare to controls. Results are shown at *p* < 0.05 FDR corrected for multiple comparisons, cluster size > 25

### Differences in static functional connectivity

**Fig. 2:**
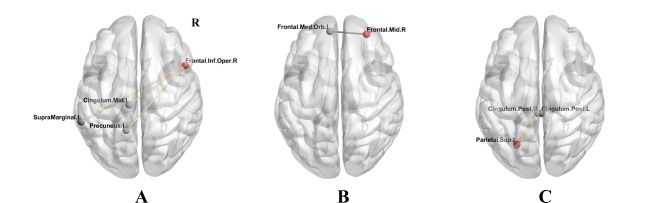
Brain regions with significantly altered functional connectivity (FC) involved in the salience network (SN) and default mode network (DMN) in the NAFLD group compared with the control group. Voxel-based independent two-sample t-test analyses were performed with a significant threshold *p* < 0.05 (FDR correction) and cluster size > 25. Red dots represent the seed point, and gray dots represent the corresponding brain regions, showing significantly altered FC with the seed point. Yellow lines represent an increased FC between the seed point and corresponding brain regions in the NAFLD group. L, left; R, right; Frontal.Inf.Oper, opercular part of inferior frontal gyrus; Cingulum.Mid, median cingulate and paracingulate gyri; SupraMarginal, supraMarginal gyrus; Frontal.Mid, middle frontal gyrus; Frontal.Med.Orb, orbital part of medial frontal gyrus; Parietal.Sup, superior parietal gyrus; Cingulum.Post, posterior cingulate gyrus

Using the right IFGoperc as the seed point, the NAFLD group had increased FC values with peak differences in the nodes of the default mode network (DMN) (left supraMarginal gyrus, left median cingulate, and paracingulate gyri and left precuneus) (Fig. 2A). Regarding the right MFG as the seed point, the NAFLD group had higher FC values with peak differences in the node of the DMN (orbital part of left medial frontal gyrus) (Fig. 2B). Regarding the left SPG as the seed point, the NAFLD group had increased FC values with peak differences at the node of the DMN (bilateral posterior cingulate gyrus (PCG)) (Fig. 2C). The detailed results are summarized in Table 4.

**Table 4:** Regions with FC significant differences in NAFLD and controls

Seed area (ROI)	Brain regions with altered functional connectivity	Cluster size (mm^3^)	Peak coordinate (MNI)			Peak *t* value
			X	Y	Z	
Right inferior frontal gyrus, opercular part (IFGoperc)	Left supraMarginal gyrus (SMG)	55	-60	-42	33	4.24
	Left median cingulate and paracingulate gyri (DCG)	26	-9	-24	42	3.58
	Left precuneus (PCUN)	25	-12	-51	63	4.36
Right middle frontal gyrus (MFG)	Left medial frontal gyrus, orbital part (MeFGorb)	43	-9	54	-12	4.26
Left superior parietal gyrus (SPG)	Bilateral posterior cingulate gyrus (PCG)	96	2	-33	31	4.14

Notes: Peak coordinate represents the locations of peak in the Montreal Neurological Institute (MNI) space and the t value means the increase (*t* > 0) or decrease (*t* < 0) of functional connectivity (FC) in NAFLD compare to controls. Results are shown at *p* < 0.05 FDR corrected for multiple comparisons, cluster size > 25

### Correlation analysis

**Fig. 3:**
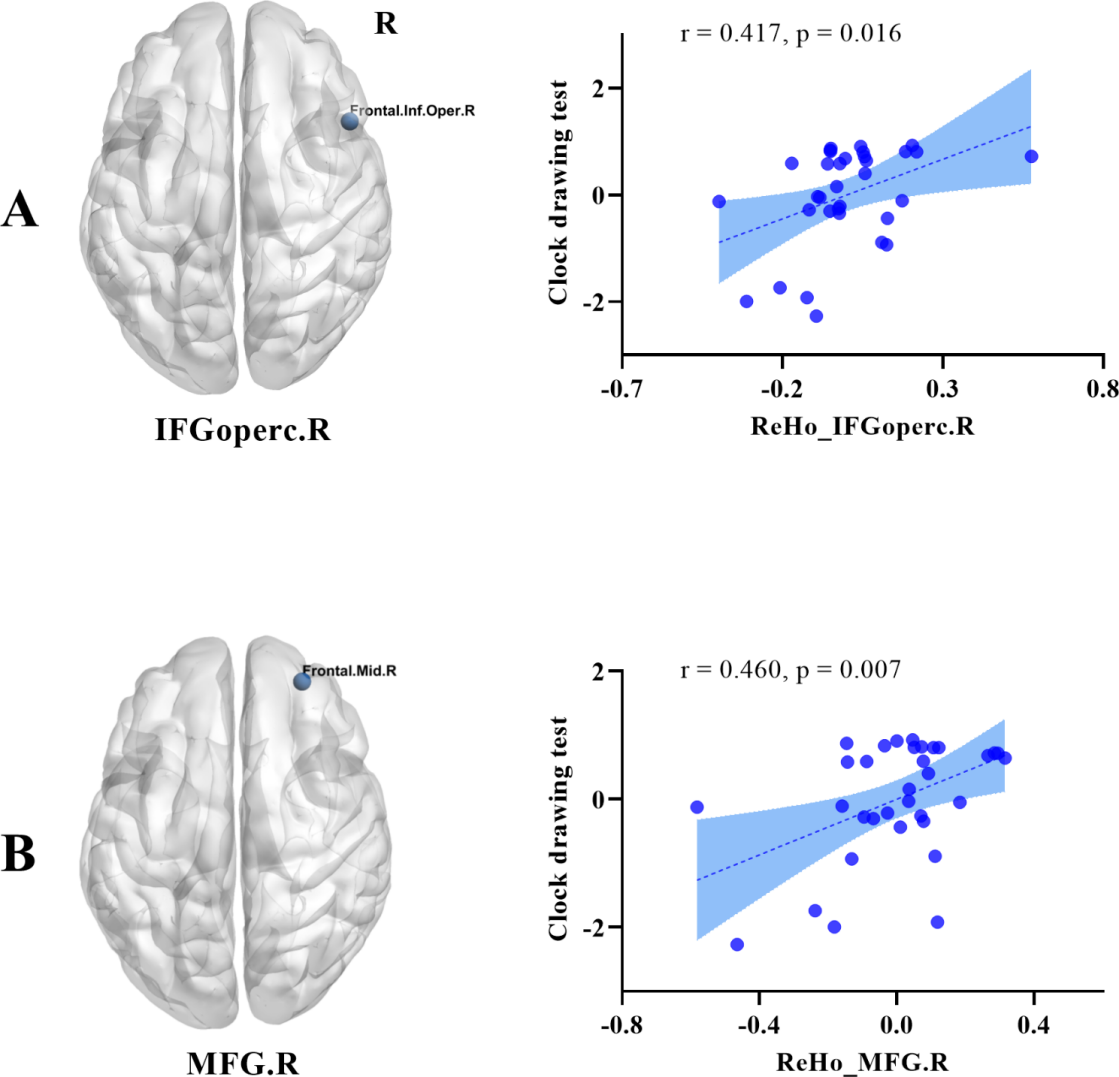
Scatter plots showing a significantly positive correlation between clock drawing test scores and ReHo values in the **(A)** right IFGoperc (*r* = 0.417, *p* = 0.016, Bonferroni corrected) and **(B)** right MFG (*r* = 0.460, *p* = 0.007, Bonferroni corrected) in the NAFLD group. The X-axis represents the residuals of the ReHo value, and the Y-axis represents the residuals of the z-transformed cognitive score, both regressing the effect of body mass index, age, and level of education. R, right; IFGoperc, opercular part of inferior frontal gyrus; MFG, middle frontal gyrus

As shown in Fig. 3, the CDT scores were moderately correlated with ReHo values in the right IFGoperc (*r* = 0.417, *p* = 0.016) and right MFG (*r* = 0.46, *p* = 0.007) in the NAFLD group. The two groups showed no other significant correlations between ReHo values and cognitive scores (Tables S2 and S3). In addition, ReHo values showed no correlation with hepatic PDFF, histological NAS, or histological NASH in the NAFLD group (Table S4).

## Discussion

This pilot study evaluated the brain neural activity and functional connectivity network in male patients with pre-cirrhosis NAFLD. This study showed that the NAFLD patients have altered regional, temporal synchronization in the right IFGoperc, right MFG, and left SPG, and increased FC involving the DMN and salience network (SN). In addition, there were significant correlations between abnormal brain neural activities and neurocognitive performance in NAFLD patients.

The prefrontal cortex was shown to be involved in spatial working memory and execution function processing [30]. Previous studies have reported dysfunction of neural activity in the frontal lobe in liver cirrhosis patients with or without hepatic encephalopathy (HE) [31, 32], which may result in cognitive deficits [32]. In this study, NAFLD patients showed abnormal ReHo in the prefrontal cortices (right IFGoperc and right MFG), suggesting that regional synchronization was altered in this brain area before developing cirrhosis in NAFLD patients. Aberrant cerebral oxygen metabolism was confirmed in advanced cirrhosis, especially in the frontal cortex. For example, CBF and cerebral glucose metabolism tended to be lower in the frontal cortex of patients with advanced cirrhosis than in controls [33, 34]. In addition, the cerebral oxygen concentration of the frontal lobe was altered in female patients with pre-cirrhosis NAFLD [14]. In conclusion, we hypothesized that this alteration in glucose metabolism might result in abnormal neural activity in the frontal regions in pre-cirrhosis NAFLD patients, thereby affecting cognition. To further investigate the brain network alterations in NAFLD, we performed seed-based (using the right IFGoperc and right MFG as seeds) FC analysis and found abnormal FC between the SN and DMN. It is believed that the SN mediates activation switching between the central executive network and DMN and that the functional integrity of the SN impacts the regulation of DMN [35]. The interaction between the SN and DMN is important for cognitive progression [36, 37]. The present study showed altered regional, temporal synchronization in the right IFGoperc and right MFG (nodes of the SN), which may contribute to SN dysfunction, lead to DMN disharmony, and further affect cognition. In addition, the aberrant ReHo values in the right IFGoperc and right MFG were significantly correlated with CDT scores, and this correlation remained even after controlling for BMI, age, and education level (Table S2 and S3), which is consistent with the findings of a previous study [30]. However, there was no significant difference in CDT scores between the two groups, most likely because patients with NAFLD were in the early stages of the disease (≤ F2). In addition, the clock drawing test is a relatively non-specific cognitive function test that involves visuospatial ability, executive function, attention, and language proficiency. It may not provide a complete picture of visuospatial function, particularly when other scales measuring visuospatial function (Rey-osterrieth Complex Figure Test) do not show significant between-group differences or other scale results do not correlate with altered ReHo values. Consequently, a study with a larger sample size is required to investigate whether abnormal neural activity in the prefrontal cortex underlies changes in visuospatial function in NAFLD. This study revealed that abnormalities in brain neural activity could be present before cognitive impairment in patients with NAFLD, highlighting the significance of early clinical intervention.

This study revealed decreased ReHo in the left SPG in NAFLD subjects compared to controls. As part of the parietal cortex, the SPG regulated information for attention and working memory [38]. Previous studies have shown altered brain neural activity in the parietal lobe region in patients with non-alcoholic cirrhosis and cirrhosis of other etiology, which was associated with disease progression, cognition, and motor dysfunction [39–41]. In addition, current evidence suggests that NAFLD can affect visuospatial function, attention, and working memory [3, 4]. In this study, abnormality of neural activity in the parietal cortex was found in NAFLD patients, which may provide new insights into the mechanism of altered attention and working memory in NAFLD. In addition, we found aberrant FC between left SPG and bilateral PCG, suggesting the involvement of DMN. DMN is important in higher-order cognition and is associated with working memory [42]. Hence, abnormal FC between these two brain regions may affect cognitive function in NAFLD.

Despite the decreased regional, temporal synchronization at two seeds, the present study also revealed that the FCs between the SN and DMN were strengthened (right MFG and left SPG). Increased connectivity between two brain regions or increased neural activity in certain regions may compensate for deficits in neuronal activity and cognitive dysfunction, which may account for unchanged cognition in many patients with early-stage NAFLD [43, 44]. In this study, we hypothesized that the increased FCs in the SN and DMN partially compensated for the decreased neural activity in the right MFG and left SPG. This compensatory response could temporarily maintain normal cognition in NAFLD. Cognitive impairments will eventually occur if reduced neural activity is not adequately compensated. In addition, weight loss was associated with the level of decrease in NAFLD severity and may be an important protective factor in NAFLD’s cognitive function during the first five years of follow-up [45, 46]. Weight loss has also positively affected the brain’s regional neural activity [47]. Therefore, if patients with NAFLD are provided with weight loss guidance (including improving diet and physical activity) at an early stage, it may not only reverse NAFLD but also prevent NAFLD patients from progressing to the stage of brain neural activity decompensation or cognitive impairment.

There were higher AVLT-immediate recall and AVLT-delayed recall scores in NAFLD patients compared to the controls in the present study. However, previous study has reported decreased AVLT scores in patients with cirrhotic patients compared to the controls [48]. These results may suggest that the performance of the memory task changes dynamically in NAFLD patients at different stages. We hypothesize that the increase in AVLT scores in the pre-cirrhotic stage of NAFLD may be a compensatory change caused by alterations of ReHo, and that the ReHo changes have a compensatory role in the maintenance of cognitive function in the NAFLD group, which needs to be further explored in future work.

There were several limitations in this study. First, the sample size of this exploratory study may limit its statistical power to detect the differences in brain neural activity between the two groups. Second, due to the study’s cross-sectional design, we could not confirm the causal relationship between the altered brain neural activity and the cognitive impairments of NAFLD. Third, only Chinese males were included, and comparable females should be recruited in future studies. Fourth, our results focused only on the resting state BOLD signal alterations. A combination of brain structure MRI, perfusion-weighted imaging, diffusion tensor imaging, and magnetic resonance spectroscopy analysis is required to enhance the reliability of the findings. Lastly, NAFLD patients with comorbid T2DM were not enrolled; thus, we were unable to determine whether T2DM has an impact on brain activity and functional connectivity in NAFLD patients. Therefore, additional longitudinal, multi-modal, gender-comparable studies with larger sample sizes involving NAFLD and its comorbidity are needed to overcome the shortcomings above.

## Conclusion

In this study, pre-cirrhosis male NAFLD subjects showed subclinical changes, including abnormal ReHo in the right IFGoperc, right MFG, and left SPG and increased FC in regions associated with the SN and DMN. In addition, altered ReHo in the prefrontal cortices was associated with cognitive function in NAFLD. Further longitudinal, multi-modal, and gender-comparable studies of large samples of NAFLD patients are required to determine if these subclinical changes are the neural basis of cognitive impairment in NAFLD.

### Electronic supplementary material

Below is the link to the electronic supplementary material.

Supplementary Material 1: Disruption of brain regional homogeneity and functional connectivity in male NAFLD: evidence from a pilot resting-state fMRI study

## Data Availability

The datasets generated and/or analyzed during the current study are not publicly available due to confidentiality but are available from the corresponding author upon reasonable request.
