# Supplementary material for: Disruption of brain regional homogeneity and functional connectivity in male NAFLD: evidence from a pilot resting-state fMRI study
Source: BMC Psychiatry. 2023 Aug 29;23:629. doi: 10.1186/s12888-023-05071-6 (PMC10463794; doi:10.1186/s12888-023-05071-6)
Supplement: Supplementary file 1 — Supplementary Material 1: Disruption of brain regional homogeneity and functional connectivity in male NAFLD: evidence from a pilot resting-state fMRI study [file 12888_2023_5071_MOESM1_ESM.docx]

**Supplementary Materials**

**Neurocognitive tests**

Neurocognitive assessments were performed by an experienced neurologist who was blinded to the subjects’ information. These scales included the following: 1) Mini-Mental State Examination (MMSE): General mental status was assessed by MMSE from six domains, including orientation, registration, attention, recall, language, and copying. An MMSE score below 27 was considered a cognitive impairment.

2) Rey-Osterreith Complex Figure Tests (CFT-Copy, CFT-Recall): The subjects were asked to copy a complex figure and then to recall the figure twenty-five to thirty minutes later. The figure was divided into 18 scoring sections, with 0 to 2 points for each section. The CFT-Copy and CFT-Recall were scored according to the same criterion.

3) Digital Span Test (DST-Forward, DST-Backward): The subjects were asked to recall the digits reported by the examiner in either a sequential or reverse order, and the DST-Forward or DST-Backward score is the maximum number of digits that could be completed by subjects.

4) Trial Making Test (TMT-A, TMT-B): The TMT-A requires subjects to connect 25 numbered circles (1-25) in ascending order, and the TMT-B requires subjects to line up 13 numbered circles (1-13) and 12 alphabetic circles (A-L) in the order that numbers and letters cross each other.

5) Clock Drawing Test (CDT): The subjects were asked to trace a clock, and the time is shown as 11:10. CDTs were scored using a five-point method and included the following four criteria (one point each): drawing the closed circle; move the digits to the correct position; include all 12 correct digits; and move the pointer into the correct position.

6) Auditory Verbal Learning Test (AVLT-Immediate recall, AVLT-Delayed recall): The subjects were required to learn a semantically categorized list of 12 concrete nouns read by the examiner, and there were two recall tests on each subject in order to acquire the AVLT scores, including AVLT-Immediate recall and AVLT-Delayed recall which separately represented the sum of the correct noun recalled by the subjects. The AVLT-Immediate recall score was the sum of the correct noun recalled by the subjects on the first recall test. After 20 min, subjects completed the second test, and the sum of the correct nouns on that test was taken as the AVLT-Delayed recall score.

| **Supplementary Table 1** Neurocognitive measurements with NAFLD compare to the Controls | | | |
| --- | --- | --- | --- |
|  | NAFLD (n=33) | Control (n=20) | *p* value |
|  |  |  |  |
| MMSE | 29 (27, 30) | 29 (28, 30) | 0.616 |
| AVLT-Immediate recall | 23 (20, 27) | 21.00 (17.25, 22.75) | **0.016*** |
| AVLT-Delayed recall | 40.79 ± 7.83 | 35.65 ± 6.68 | **0.018*** |
| CDT | 3 (3, 4) | 3 (4, 4) | 0.818 |
| TMT-A | 39.97 ± 13.94 | 47.20 ± 20.17 | 0.233 |
| TMT-B | 113 (88, 177) | 130 (96, 163) | 0.367 |
| CFT-Copy | 36 (34, 36) | 36 (35, 36) | 0.946 |
| CFT-Recall | 20.52 ± 8.07 | 16.50 ± 7.13 | 0.073 |
| DST-Forward | 8 (7, 8) | 6.50 (5.25, 9.00) | 0.146 |
| DST-Backward | 4 (4, 5) | 5.00 (4.00, 5.75) | 0.337 |
| Notes: Values are mean (± standard deviation) or median (interquartile range) Abbreviations: MMSE, Mini-Mental State Examination; AVLT, Auditory Verbal Learning Test; CDT, Clock Drawing Test; TMT, Trail Making Test; CFT, Rey-osterrieth Complex Figure Test; DST, Digital Span Test | | | |

| **Supplementary Table 2** Relationship between the ReHo value and Z-transformed cognitive score in the two groups (adjusted for covariates) | | | | | | | | | | | |
| --- | --- | --- | --- | --- | --- | --- | --- | --- | --- | --- | --- |
| Brain Region | Group | MMSE | | AVLT-Immediate recall | | AVLT-Delayed recall | | CDT | | TMT-A | |
|  |  | r^a^ | p | r^a^ | p | r^a^ | p | r^a^ | p | r^a^ | p |
| Right inferior frontal gyrus, opercular part | NAFLD | 0.250 | 0.160 | -0.047 | 0.797 | -0.064 | 0.723 | 0.417 | **0.016*** | -0.246 | 0.168 |
|  | Control | 0.034 | 0.886 | -0.259 | 0.270 | -0.223 | 0.346 | 0.230 | 0.329 | -0.296 | 0.205 |
| Right middle frontal gyrus | NAFLD | 0.260 | 0.143 | 0.109 | 0.547 | 0.104 | 0.565 | 0.460 | **0.007*** | 0.071 | 0.696 |
|  | Control | 0.076 | 0.750 | -0.224 | 0.342 | -0.181 | 0.445 | 0.169 | 0.476 | -0.253 | 0.282 |
| Left superior parietal gyrus | NAFLD | 0.080 | 0.660 | 0.209 | 0.243 | -0.077 | 0.669 | -0.133 | 0.459 | 0.001 | 0.996 |
|  | Control | 0.055 | 0.818 | 0.327 | 0.159 | 0.370 | 0.108 | -0.282 | 0.228 | -0.176 | 0.459 |
| Notes: Bonferroni correction was performed to multiple comparison. P <0.017 (0.05/3) was considered as a significant difference Abbreviations: MMSE, Mini-Mental State Examination; AVLT, Auditory Verbal Learning Test; CDT, Clock Drawing Test; TMT, Trail Making Test; CFT, Rey-osterrieth Complex Figure Test; DST, Digital Span Test | | | | | | | | | | | |

| **Supplementary Table 2 (Continued)** | | | | | | | | | | | |
| --- | --- | --- | --- | --- | --- | --- | --- | --- | --- | --- | --- |
| Brain Region | Group | TMT-B | | CFT-Copy | | CFT-Delay | | DST-Forward | | DST-Backward | |
|  |  | r^a^ | p | r^a^ | p | r^a^ | p | r^a^ | p | r^a^ | p |
| Right inferior frontal gyrus, opercular part | NAFLD | -0.366 | 0.036 | -0.029 | 0.874 | 0.017 | 0.923 | 0.006 | 0.974 | 0.011 | 0.951 |
|  | Control | 0.292 | 0.226 | 0.141 | 0.552 | 0.248 | 0.291 | -0.403 | 0.078 | -0.094 | 0.693 |
| Right middle frontal gyrus | NAFLD | -0.070 | 0.699 | 0.007 | 0.967 | 0.118 | 0.512 | -0.033 | 0.855 | 0.176 | 0.329 |
|  | Control | 0.250 | 0.302 | 0.149 | 0.530 | -0.123 | 0.606 | 0.080 | 0.739 | 0.252 | 0.283 |
| Left superior parietal gyrus | NAFLD | 0.358 | 0.041 | -0.110 | 0.540 | 0.026 | 0.886 | 0.154 | 0.392 | 0.232 | 0.195 |
|  | Control | -0.457 | 0.049 | -0.066 | 0.782 | -0.219 | 0.353 | 0.400 | 0.081 | 0.037 | 0.876 |
| Notes: Bonferroni correction was performed to multiple comparison. P <0.017 (0.05/3) was considered as a significant difference Abbreviations: MMSE, Mini-Mental State Examination; AVLT, Auditory Verbal Learning Test; CDT, Clock Drawing Test; TMT, Trail Making Test; CFT, Rey-osterrieth Complex Figure Test; DST, Digital Span Test | | | | | | | | | | | |

| **Supplementary Table 3** Relationship between the ReHo value and Z-transformed cognitive score in the two groups (unadjusted for covariates) | | | | | | | | | | | |
| --- | --- | --- | --- | --- | --- | --- | --- | --- | --- | --- | --- |
| Brain Region | Group | MMSE | | AVLT-Immediate recall | | AVLT-Delayed recall | | CDT | | TMT-A | |
|  |  | r^a^ | p | r^a^ | p | r^a^ | p | r^a^ | p | r^a^ | p |
| Right inferior frontal gyrus, opercular part | NAFLD | 0.178 | 0.323 | 0.014 | 0.937 | -0.077 | 0.670 | 0.423 | **0.014*** | -0.164 | 0.361 |
|  | Control | 0.210 | 0.373 | 0.008 | 0.974 | 0.003 | 0.990 | 0.328 | 0.158 | -0.258 | 0.273 |
| Right middle frontal gyrus | NAFLD | 0.273 | 0.124 | 0.223 | 0.212 | 0.132 | 0.463 | 0.475 | **0.005*** | 0.030 | 0.869 |
|  | Control | 0.177 | 0.456 | -0.079 | 0.741 | -0.056 | 0.815 | 0.155 | 0.513 | -0.395 | 0.085 |
| Left superior parietal gyrus | NAFLD | 0.107 | 0.554 | 0.198 | 0.270 | 0.044 | 0.808 | -0.105 | 0.560 | 0.018 | 0.920 |
|  | Control | -0.072 | 0.764 | 0.107 | 0.652 | 0.193 | 0.416 | -0.373 | 0.105 | -0.238 | 0.313 |
| Notes: Bonferroni correction was performed to multiple comparison. P <0.017 (0.05/3) was considered as a significant difference  Abbreviations: MMSE, Mini-Mental State Examination; AVLT, Auditory Verbal Learning Test; CDT, Clock Drawing Test; TMT, Trail Making Test; CFT, Rey-osterrieth Complex Figure Test; DST, Digital Span Test | | | | | | | | | | | |

| **Supplementary Table 3 (Continued)** | | | | | | | | | | | |
| --- | --- | --- | --- | --- | --- | --- | --- | --- | --- | --- | --- |
| Brain Region | Group | TMT-B | | CFT-Copy | | CFT-Delay | | DST-Forward | | DST-Backward | |
|  |  | r^a^ | p | r^a^ | p | r^a^ | p | r^a^ | p | r^a^ | p |
| Right inferior frontal gyrus, opercular part | NAFLD | -0.309 | 0.080 | -0.065 | 0.717 | 0.011 | 0.951 | 0.009 | 0.960 | -0.035 | 0.848 |
|  | Control | 0.167 | 0.493 | 0.231 | 0.327 | 0.377 | 0.101 | -0.244 | 0.300 | -0.025 | 0.916 |
| Right middle frontal gyrus | NAFLD | -0.201 | 0.262 | 0.010 | 0.956 | 0.111 | 0.539 | 0.051 | 0.779 | 0.077 | 0.670 |
|  | Control | -0.075 | 0.760 | 0.290 | 0.215 | 0.070 | 0.770 | 0.250 | 0.288 | 0.371 | 0.107 |
| Left superior parietal gyrus | NAFLD | 0.234 | 0.191 | -0.048 | 0.791 | -0.031 | 0.862 | 0.142 | 0.429 | 0.006 | 0.974 |
|  | Control | -0.414 | 0.078 | 0.131 | 0.582 | -0.118 | 0.621 | 0.372 | 0.107 | 0.006 | 0.981 |
| Notes: Bonferroni correction was performed to multiple comparison. P <0.017 (0.05/3) was considered as a significant difference Abbreviations: MMSE, Mini-Mental State Examination; AVLT, Auditory Verbal Learning Test; CDT, Clock Drawing Test; TMT, Trail Making Test; CFT, Rey-osterrieth Complex Figure Test; DST, Digital Span Test | | | | | | | | | | | |

| **Supplementary Table 4** Relationship between the ReHo value and liver pathology in the NAFLD group | | | | | | | |
| --- | --- | --- | --- | --- | --- | --- | --- |
| Brain Region | Covariates* | NAS | | NASH | | Liver MRI-PDFF | |
|  |  | r^a^ | p | r^a^ | p | r^a^ | p |
| Right inferior frontal gyrus, opercular part | adjusted | -0.302 | 0.087 | -0.276 | 0.119 | -0.004 | 0.981 |
|  | unadjusted | -0.268 | 0.131 | -0.228 | 0.202 | -0.025 | 0.891 |
| Right middle frontal gyrus | adjusted | -0.12 | 0.506 | -0.154 | 0.392 | -0.136 | 0.449 |
|  | unadjusted | -0.092 | 0.611 | -0.08 | 0.658 | -0.103 | 0.569 |
| Left superior parietal gyrus | adjusted | 0.031 | 0.864 | 0.018 | 0.919 | 0.088 | 0.628 |
|  | unadjusted | 0.097 | 0.59 | 0.121 | 0.503 | 0.193 | 0.282 |
| Notes:   1. * Body mass index, age, and education was regressed out as covariates  2. Bonferroni correction was performed to multiple comparison. P <0.017 (0.05/3) was considered as a significant difference Abbreviations: NAS, NAFLD activity score; NASH, non-alcoholic steatohepatitis; PDFF, proton density fat fraction | | | | | | | |
